# Changes in Gait Self-Efficacy, Fear of Falls, and Gait Four and Eight Months after Bariatric Surgery

**DOI:** 10.3390/bs12080246

**Published:** 2022-07-22

**Authors:** Danny Shin, Laura Keegan, Simone V. Gill

**Affiliations:** 1 Department of Occupational Therapy, Boston University, Boston, MA 02215, USA; ddshin@bu.edu (D.S.); lakeegan@bu.edu (L.K.); 2 Department of Medicine, Boston University, Boston, MA 02215, USA; 3 Department of Psychological and Brain Sciences, Boston University, Boston, MA 02215, USA

**Keywords:** obesity, bariatric surgery, self-efficacy, fear of falling, gait

## Abstract

After bariatric surgery, individuals improve walking characteristics related to fall risk. However, little is known about psychosocial factors, such as gait self-efficacy and fear of falling, after surgery. Our objectives were to (1) examine how weight loss affects psychosocial factors and gait four and eight months after bariatric surgery, as well as (2) determine if there is a relationship between gait self-efficacy and fear of falling. Fourteen adults scheduled to undergo bariatric surgery completed three visits: before surgery, four and eight months after surgery. Gait self-efficacy was measured with the Modified Gait Efficacy Scale, and fear of falls was measured with the Tinetti Falls Efficacy Scale. Gait measures were collected during five conditions: initial baseline and final baseline on flat ground, and crossing obstacles of three heights. Gait self-efficacy or fear of falling did not change after surgery. However, both four and eight months after surgery, higher gait self-efficacy and lower fear of falling were correlated with longer and faster steps during all conditions (all *p*s < 0.05). Focusing interventions on psychosocial measures related to gait may yield longer lasting improvements in walking after surgery, ultimately resulting in a decreased fall risk and higher quality of life.

## 1. Introduction

Obesity is associated with chronic health conditions such as osteoarthritis and heart disease, which impact function and independence [1,2]. The combination of excess body mass and symptoms of chronic conditions including joint pain and decreased cardiovascular endurance lead to impairments in walking, subsequent fall risks, and decreased participation in physical activity. Clinical measurements like the Functional Gait Assessment [3] and biomechanical instrumentation [4] reveal balance impairments in people with obesity compared to their normal weight peers. Adults with obesity tend to walk more slowly, and take shorter steps [5]. Along with chronic health conditions and impairments in motor function, psychosocial factors co-exist with obesity: anxiety [6], poor body-image [7], and depression [6], resulting in a reduced quality of life [2]. Thus, it is important to use an interdisciplinary approach to examine physical and psychosocial factors affecting this population [8].

Bariatric surgery is a safe and effective way to combat morbid obesity by facilitating significant weight loss; patients lose up to 35% of their body weight within the first year [9]. Weight loss after surgery improves motor function by decreasing the amount of time spent with both feet on the ground [10], increasing speed and the length [11] of patients’ steps. These changes result in patients’ improved ability to safely complete functional tasks such as crossing obstacles in their walking paths [10]. Psychosocial factors, such as depressive symptoms and anxiety and self-esteem [12], also improve after surgery [13]. Unfortunately, up to 25% of patients are unable reach the weight loss goals necessary to yield the substantial benefits of bariatric surgery [14] and regain 23% of the weight lost within seven years [15].

Two main psychosocial factors affect patients’ ability to lose and maintain weight loss: self-efficacy and fear of falling. Low motivation and self-efficacy to regularly perform physical activity are known to be barriers to long-term weight loss [16,17]. Unsuccessful weight loss outcomes after surgery have been linked to patients’ feelings of low self-efficacy in making dietary changes [18], which improves somewhat after surgery for some patients [19]. In other populations, self-efficacy is related to motor function and physical activity; older adults with low gait self-efficacy engage in less community mobility regardless of their level of motor functioning [20]. The same is true for fear of falling, which is present in adult woman and is associated with low levels of physical activity [21]. Whether self-efficacy and fear of falling impact gait in patients with obesity after bariatric surgery is still unknown.

The aims of this study were twofold: (1) to examine changes in patients’ gait self-efficacy, fear of falling, and motor function four and eight months after bariatric surgery. We hypothesized that gait self-efficacy would increase and that fear of falling would decrease four months after surgery. We also projected that patients would experience continued increases in gait self-efficacy and decreases in fear of falling eight months after surgery and (2) to determine if there is a relationship between gait self-efficacy and fear of falling and gait measures. We hypothesized that gait self-efficacy would be positively correlated and fear of falling would be negatively correlated with gait measures after bariatric surgery.

## 2. Materials and Methods

### 2.1. Participants

Fourteen adults (10 women, Mean_age_ = 48.36 years old, *SD* = 12.28, BMI = 42.45 kg/m^2^, *SD* = 4.66) scheduled to undergo Roux-en-Y bariatric surgery were recruited from the Massachusetts General Hospital Weight Center. Study criteria included being between the ages 30–60 years old, ability to speak and read English, and ability to walk independently without an assistive device. Participants were excluded if they had or were scheduled for knee surgery; were receiving dialysis; being treated for cancer; and had significant cardiovascular, musculoskeletal, vestibular, or neurologic conditions.

### 2.2. Gait Measures

Dynamic balance was assessed with the Functional Gait Assessment (FGA). The FGA is a 10-item tool used to assess balance while performing walking tasks such as stepping over an obstacle, making a turn, and walking with feet close together. Performance on items are graded on a 4-level ordinal scale, higher scores indicate better performance, with a maximum possible score of 30. The FGA has demonstrated high reliability, internal consistency and validity [22] across many clinical populations [3,23]. 

A 6.10 m long × 0.89 m wide pressure-sensitive gait carpet (Protokinetics, LLC; Peekskill, NY, USA) was used to collect spatiotemporal variables during a gait task. As participants walked down the gait carpet, pressure from their footfalls activated piezoelectric sensors to compute distance (x and y coordinates) and timing of steps. Spatiotemporal gait variables were processed with the Protokinetics Movement Analysis Software (version 5.08c2). The dependent variables collected were how long (step length, cm), wide (step width, cm), percentage of time spent on two feet during the gait cycle (double limb support percentage, % of the gait cycle), and fast (velocity, cm/s) steps were during walking conditions.

### 2.3. Psychosocial Measures

Two measures were used to assess self-efficacy in gait and falls. The Modified Gait Efficacy Scale (MGES) scale is a 10-item self-report measure designed to measure confidence in walking without falling under everyday circumstances. Items are scored on a 10-point Likert scale from 1 (“no confidence”) to 10 (“complete confidence”). Everyday circumstances include: walking on a level surface, stepping over an obstacle, stepping up and down from a curb, walking up and down stairs with handrails and without handrails, and walking long distances. This measure has established high reliability, validity and consistency [20] across a variety of conditions [24,25].

The Tinetti Falls Efficacy Scale (TFES) is a 10-item questionnaire that assesses confidence in participants’ perceived ability to perform daily tasks without falling. Items on the questionnaire include: taking a bath or shower, reaching into cabinets or closets, preparing meals not requiring heavy or hot objects, walking around the house, getting in and out of bed, answering the door or telephone, getting in and out of a chair, getting dressed and undressed, personal grooming and getting on and off a toilet. Items are scored on a 10-point Likert scale from 1 (“very confident”) to 10 (“not confident at all”). This measure has high reliability, validity and consistency across clinical populations [26,27].

### 2.4. Procedure

Participants visited the Motor Development Lab for 3 visits: before bariatric surgery, 4 months after surgery, and 8 months after surgery. After providing consent, participants’ height, and weight were measured with a stadiometer and scale respectively. Next, a trained experimenter administered the FGA. Physical demonstrations were provided when necessary. After the FGA, participants completed the MGES and TFES, and scores were computed. Then, participants walked at a self-selected pace on the gait carpet for a total of 50 trials. The 50 trials included five conditions of 10 trials each: initial baseline on flat ground, crossing three separate obstacle heights (low, medium and high), and final baseline on flat ground. Obstacles were created by placing a wooden dowel (121 cm long) into different holes (4 cm for low, 8 cm for medium, and 16 cm for high) on the vertical face of two wooden rectangular towers. Obstacle heights represented the height of impediments encountered during everyday walking such as curb or stairs. Obstacle heights were counterbalanced across visits and participants.

### 2.5. Sample Size

The sample size was estimated for changes in our primary endpoint: gait spatiotemporal variables (e.g., velocity). The sample size for gait changes post-surgery was estimated from Vincent et al. [11] in which a 15% increase (0.162 ± 0.15 m/s) in velocity was observed in 25 patients post-surgery. With this model, 9 subjects are needed to observe significantly improved gait parameters. We assume a standard deviation (SD) of 20 m/s and a Pearson’s correlation of 0.6 between the two visits; this implies an SD of change equal to about 18 m/s. Under these assumptions, our design will obtain in excess of 90% power to detect between-group differences of 15.2 m/s or greater. 

### 2.6. Statistical Analyses

All statistical analyses were conducted with SPSS (Version 26.0, SPSS Inc., Chicago, IL, USA). Descriptives were run for all variables. In order to investigate differences in baseline conditions, a paired sample t-test was used to compare initial and final baseline conditions. Prior to running repeated measure (RM) analysis of variance (ANOVA), data were examined to ensure that there were no violations of statistical assumptions. One-way RM ANOVAs with study visit as the independent variable were run on the FGA, spatiotemporal and psychosocial measures. Pearson’s correlations were run to examine the relationships between the physical factors (FGA and spatiotemporal gait variables), psychosocial factors (MGES and TFES) and between physical and psychosocial factors at all three study visits. To examine if there were changes in gait while crossing obstacles, we ran 3 (study visit) × 3 (obstacle height) RM ANOVAs on spatiotemporal measures. Post hoc analyses included pairwise comparisons. Bonferroni corrections was used for all tests.

## 3. Results

### 3.1. Psychosocial Outcomes

We found no main effect for visit on MGES and TFES scores; participants did not experience a change in gait self-efficacy or fear of falling after surgery (Table 1). Before surgery, psychosocial variables were not correlated with spatiotemporal gait measures. Four months after surgery, higher gait self-efficacy scores were correlated with longer steps during flat ground walking and during obstacle conditions. The results showed that MGES scores were positively correlated with step length at the low obstacle (r(13) = 0.62, *p* < 0.05), medium obstacle (r(13) = 0.56, *p* < 0.05), high obstacle (r(13) = 0.64, *p* < 0.05), and final baseline (r(13) = 0.61, *p* < 0.05) conditions. Four months after surgery, higher gait self-efficacy was correlated with faster steps during flat ground walking and during obstacle conditions. Findings revealed that MGES scores were positively correlated with velocity at the low obstacle (r(13) = 0.61, *p* < 0.05), medium obstacle (r(13) = 0.57, *p* < 0.05), high obstacle (r(13) = 0.62, *p* < 0.05) and final baseline (r(13) = 0.64, *p* < 0.05) conditions. 

**Figure 1:**
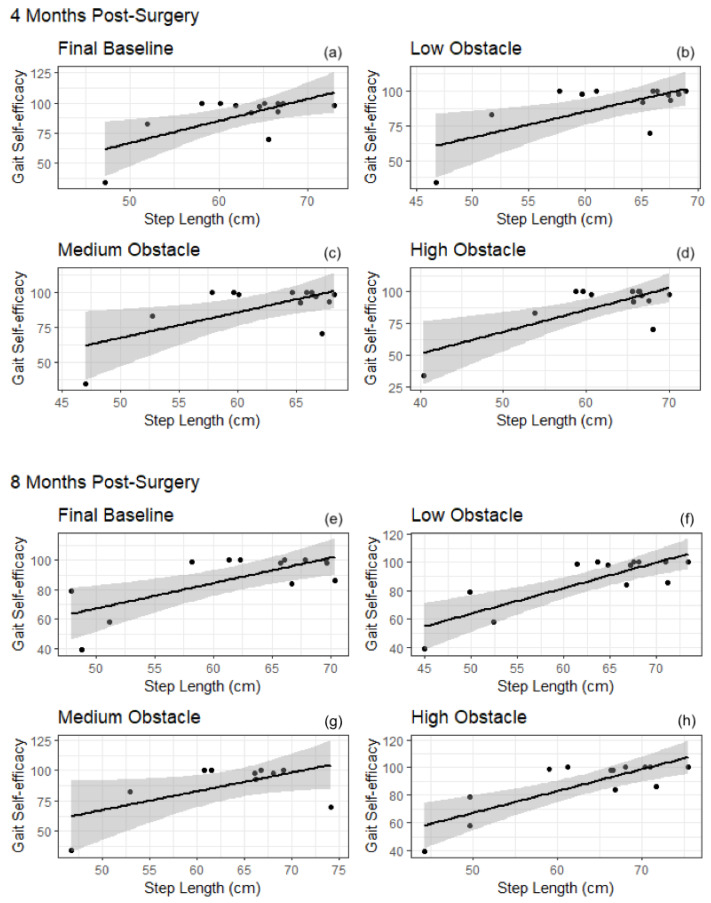
Correlations between gait self-efficacy and step length for final baseline (**a**,**e**), low obstacle (**b**,**f**), medium obstacle (**c**,**g**), and high obstacle conditions (**d**,**h**) after bariatric surgery. Gait self-efficacy were significantly associated with step length in all conditions. A general linear model was used to fit the trend lines. Clouds represent 95% CI.

**Figure 2:**
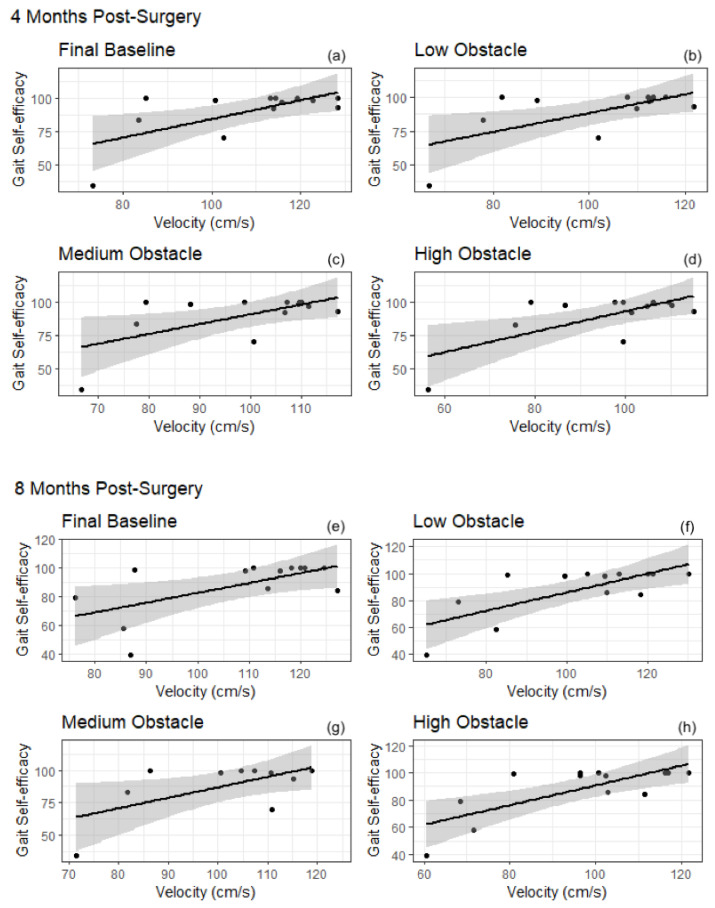
Correlations between gait self-efficacy and velocity for final baseline (**a**,**e**), low obstacle (**b**,**f**), medium obstacle (**c**,**g**) and high obstacle conditions (**d**,**h**) after bariatric surgery. Gait self-efficacy were significantly associated with velocity in all conditions. A general linear model was used to fit the trend lines. Clouds represent 95% CI.

Eight months after surgery, higher gait self-efficacy scores were correlated with longer steps during flat ground walking and obstacle conditions (Figure 1); MGES scores were positively correlated with step length during initial baseline (r(13) = 0.70, *p* < 0.05), low obstacle (r(13) = 0.82, *p* < 0.005), medium obstacle (r(13) = 0.78, *p* < 0.005), high obstacle (r(13) = 0.80, *p* < 0.005) and final baseline (r(13) = 0.72, *p* < 0.05) conditions. Higher gait self-efficacy scores were correlated with faster steps during flat ground walking and obstacle conditions (Figure 2). The results demonstrated that MGES scores were positively correlated with velocity at initial baseline (r(13) = 0.58, *p* < 0.05), low obstacle (r(13) = 0.73, *p* < 0.05), medium obstacle (r(13) = 0.78, *p* < 0.005), high obstacle (r(13) = 0.74, *p* < 0.005) and final baseline (r(13) = 0.61, *p* < 0.05) conditions.

Four months after surgery, lower fear of falls was correlated with longer steps during flat ground walking and obstacle conditions. The results showed that TFES scores were negatively correlated with step length at initial baseline (r(13) = −0.57, *p* < 0.05), low obstacle (r(13) = −0.67, *p* < 0.05), medium obstacle (r(13) = −0.67, *p* < 0.05), high obstacle conditions (r(13) = −0.80, *p* < 0.005) and final baseline (r(13) = −0.65, *p* < 0.05). Lower fear of falls was also correlated with faster steps during flat ground walking and obstacle conditions. TFES scores were correlated with velocity at low obstacle (r(13) = −0.59, *p* < 0.05), medium obstacle(r(13) = −0.59, *p* < 0.05), high obstacle (r(13) = −0.68, *p* < 0.05) and final baseline (r(13) = −0.57, *p* < 0.05) conditions. 

Eight months after surgery, lower fear of falls was correlated with longer steps during obstacle conditions. TFES scores were negatively correlated with step length at low obstacle (r(13) = −0.65, *p* < 0.05), medium obstacle (r(13) = −0.63, *p* < 0.05), high obstacle (r(13) = −0.63, *p* < 0.05) conditions. 

### 3.2. Flat Ground Walking and Balance

There were no significant differences found in spatiotemporal variables between initial and final baseline within visits (*p* > 0.05). Therefore, variables at initial baseline were used for subsequent analysis of flat ground walking. 

We found a main effect for visit on FGA (F(1.16, 16.27) = 6.25, partial *η*^2^ = 0.31, *p* < 0.05), step width (F(2, 30) = 12.58, partial *η*^2^ = 0.46, *p* < 0.001), step length (F(1.41, 21.19) = 4.86, partial *η*^2^ = 0.25, *p* < 0.05), double limb support percentage (F(2, 30) = 9.59, partial *η*^2^ = 0.39, *p* < 0.005), and velocity (F(2, 30) = 4.52, partial *η*^2^ = 0.23, *p* < 0.05). Post hoc comparisons showed that patients walked with narrower steps 4 months after surgery and 8 months after surgery. Patients spent less time in double limb support 8 months after surgery (all *p*s < 0.01). Post hoc comparisons showed no significant changes in FGA, step length and velocity following Bonferroni corrections (all *p*s > 0.01).

### 3.3. Obstacle Crossing

We found a main effect for visit on step width (F(1.14, 17.11) = 8.11, partial *η*^2^ = 0.35, *p* < 0.05), step length (F(1.18, 17.68) = 6.53, partial *η*^2^ = 0.30, *p* < 0.05), double limb support percentage (F(1.36, 20.36) = 5.45, partial *η*^2^ = 0.27, *p* < 0.05) and velocity (F(1.31, 19.68) = 6.74, partial *η*^2^ = 0.31, *p* < 0.05). Patients walked faster from 4 months to 8 months after surgery (*p* < 0.01). Post hoc comparisons showed no significant change in step width, step length, and double support percentage between visits following Bonferroni corrections.

There was also a main effect for obstacle height on double support percentage (F(1.56, 23.46) = 30.50, partial *η*^2^ = 0.67, *p* < 0.001, and velocity (F(1.63, 24.49) = 64.49, partial *η*^2^ = 0.80, *p* < 0.001). Patients spent less time on both feet and walked more slowly as obstacle heights increased from low to medium and low to high heights (all *p*s < 0.01).

There were no significant interactions between visit and obstacle height for any of the spatiotemporal variables.

## 4. Discussion

The purpose of this study was to examine whether gait self-efficacy and fear of falling would change after weight loss following bariatric surgery. We found no significant changes in gait self-efficacy and fear of falling scores after weight loss surgery. Additionally, we aimed to determine if there was a correlation between the two psychosocial measures (gait self-efficacy and fear of falling) and gait. There was no correlation between the psychosocial measures and gait before surgery. Interestingly, we found that gait self-efficacy was positively correlated with step length and velocity four and eight months after bariatric surgery during flat ground walking and while crossing obstacles. Fear of falling was negatively correlated with step length and velocity during flat ground walking and while crossing obstacles. Eight months after surgery, fear of falling was negatively correlated with step length only while crossing obstacles. Only after participants lost weight through surgery did gait self-efficacy and fear of falling became more influential on walking characteristics. This finding illustrates that psychosocial variables related to gait may be avenues for intervention after surgery, but not before.

As expected, patients’ balance and gait measures improved four and eight months after surgery. This finding is consistent with separate studies reporting improvements in gait three months [11] and eight months after surgery [28]. Currently, rehabilitation services, like occupational and physical therapy, are only recommended due to decreases in functional status 12 months after surgery [29]. As stabilization of weight loss occurs one-year post surgery [14], it may be beneficial to implement interventions to maximize gait improvement prior to their annual follow-up. Based on our knowledge, this is the first study to investigate gait following surgery at two distinct time points (four and eight months) within the same cohort during this crucial one-year recovery period. 

Contrary to our hypothesis, we found no differences in gait self-efficacy or fear of falling after surgery. Although self-efficacy for behavioral modification and general self-efficacy is well studied in bariatric surgery recipients [19,30], it was unknown whether candidates of bariatric surgery have lower self-efficacy specific to gait before or after their surgically induced weight loss. We found that patients had high gait self-efficacy before bariatric surgery. It is possible that before surgery, although participants displayed gait characteristics associated with falls, they were confident in using wider, shorter, and slower steps to walk while performing everyday activities. Interestingly, participants continued to demonstrate high gait self-efficacy after experiencing weight loss after their walking improved. One way to improve self-efficacy is to experience success after engaging in an activity [31]. In our sample, since testing occurred four and eight-months after surgery, patients may have experienced adequate success in walking thereby linking gait self-efficacy with movement outcomes. This finding may provide evidence for intervening before four months after surgery to maximize subsequent gains in gait self-efficacy.

Similar to gait self-efficacy, we found no change in fear of falling over our three time points. Patients with obesity have a 31% higher risk of falling [32]. However, our patients had a low fear or falling across the three study visits. Four months after surgery, there was a negative correlation between fear of falling and two gait measures: step length and velocity; lower fear of falling was linked with faster and longer steps. Eight months after surgery, fear of falling was negatively correlated with step length. As walking improved, patients may have become less fearful while walking.

Current clinical guidelines define the primary care team as the bariatric surgeon, obesity specialist, and dietitian [33]. Our findings add to the growing body of work to include additional members to the multidisciplinary care team. Some recipients of bariatric surgery demonstrate new eating disorders [34], difficulty maintaining nutritional and exercise guidelines [35], leading to weight regain. Including occupational therapists, mental health professionals to address these psychosocial factors may help all bariatric patients experience a positive outcome of their surgery.

Future research may focus on investigating the effect of bariatric surgery on additional psychosocial factors, such as depression and anxiety, and their relationship to gait. This would provide further rationale to diversify the primary care team for bariatric patients. In addition, further research may be conducted at time points closer to when weight loss stabilizes (around 12 months). This would help screen patients that may require additional services to maximize the results of their bariatric surgery.

### Limitations

Our study was not without limitations. First, there were more female than male participants in our study. However, almost 80% of the bariatric recipients in the past decade were female [36], allowing the results of our study be generalized to this specific population. Second, we only included those with the ability to independently walk without an assistive device, which prevented people with a higher risk of falls from entering our study. Nevertheless, our sample includes participants who may encounter more falls due to having the opportunity to walk independently and possibly more frequently. Additionally, using psychosocial measures related to falls in people who can independently walk may have created a ceiling effect. Third, there was a large variability in the age of our participants. Still, we believe our findings are representative of people that undergo bariatric surgery. Last, anthropomorphic changes such as thigh circumference were not measured. 

## 5. Conclusions

Gait self-efficacy and fear of falling did not change after bariatric surgery but were correlated with gait during flat ground and obstacle crossing after bariatric surgery. Interventions targeting psychosocial measures may yield longer lasting improvement in walking, ultimately resulting reduced fall risk and higher quality of life. Finding relationships between psychosocial and gait patterns associated with falling may inform interventions to bolster walking ability and reduce fall risk in this population.

## Figures and Tables

**Table 1:** Descriptives and spatiotemporal gait parameters during initial baseline walking across visits and assessment scores. Standard Deviation scores are in parentheses.

	Pre-Surgery	4 Months Post-Surgery	8 Months Post-Surgery
**Height (cm)**	166.36 (11.79)	166.36 (11.79)	166.36 (11.79)
**Weight (kg)**	120.04 (24.52)	95.68 (21.90) *	90.78 (21.96) *
**BMI (kg/m^2^)**	42.95 (4.01)	34.64 (4.07) *	32.87 (4.53) *
**Step Width (cm)**	10.87 (3.72)	8.60 (3.17) *	8.21 (2.93) *
**Step Length (cm)**	59.43 (7.58)	61.18 (7.22)	63.09 (7.40) *
**Double Support %**	35.68 (4.77)	33.87 (3.83)	32.41 (2.62)
**Velocity (cm/s)**	101.85 (19.85)	104.69 (16.69)	110.90 (17.47)
**FGA ^1^**	23.93 (6.26)	26.21 (4.81)	27.00 (4.79)
**MGES ^2^**	92.43 (7.64)	91.31 (13.76)	87.54 (19.88)
**TFES ^3^**	12.07 (3.54)	12.38 (7.43)	10.83 (5.22)

^1^ Functional Gait Assessment ^2^ Modified Gait Efficacy Scale ^3^ Tinetti Falls Efficacy Scale. * Denotes significant difference from before surgery.

## Data Availability

The data presented in this study are available on request from the corresponding author. The data are not publicly available due to the consent provided by participants on the use of confidential data.
